# The Human Proteins MBD5 and MBD6 Associate with Heterochromatin but They Do Not Bind Methylated DNA

**DOI:** 10.1371/journal.pone.0011982

**Published:** 2010-08-06

**Authors:** Sophie Laget, Michael Joulie, Florent Le Masson, Nobuhiro Sasai, Elisabeth Christians, Sriharsa Pradhan, Richard J. Roberts, Pierre-Antoine Defossez

**Affiliations:** 1 New England Biolabs, Ipswich, Massachusetts, United States of America; 2 CNRS UMR7216, Université Paris-Diderot, Paris, France; 3 CNRS UMR 5547, Université Toulouse 3, Toulouse, France; National Institute on Aging, United States of America

## Abstract

**Background:**

MBD5 and MBD6 are two uncharacterized mammalian proteins that contain a putative Methyl-Binding Domain (MBD). In the proteins MBD1, MBD2, MBD4, and MeCP2, this domain allows the specific recognition of DNA containing methylated cytosine; as a consequence, the proteins serve as interpreters of DNA methylation, an essential epigenetic mark. It is unknown whether MBD5 or MBD6 also bind methylated DNA; this question has interest for basic research, but also practical consequences for human health, as *MBD5* deletions are the likely cause of certain cases of mental retardation.

**Principal Findings:**

Here we report the first functional characterization of MBD5 and MBD6. We have observed that the proteins colocalize with heterochromatin in cultured cells, and that this localization requires the integrity of their MBD. However, heterochromatic localization is maintained in cells with severely decreased levels of DNA methylation. *In vitro*, neither MBD5 nor MBD6 binds any of the methylated sequences DNA that were tested.

**Conclusions:**

Our data suggest that MBD5 and MBD6 are unlikely to be methyl-binding proteins, yet they may contribute to the formation or function of heterochromatin. One isoform of MBD5 is highly expressed in oocytes, which suggests a possible role in epigenetic reprogramming after fertilization.

## Introduction

DNA methylation is an essential epigenetic mark in mammals. It regulates the expression of imprinted genes, and possibly also non-imprinted genes. It maintains the repression of the inactive X in female mammals. Finally, it is essential to ensure the transcriptional silencing of repeated sequences [1], [2].

Nine different proteins are currently known to bind methylated DNA in mammals; they are called Methyl-Binding Proteins (MBPs), and they fall into three structural families [3]. The first family contains MBD1, MBD2, MBD4, and MeCP2; these proteins share a domain called methyl-CpG binding domain (MBD). The second family comprises UHRF1 and UHRF2, which bind methylated DNA via a SET- and Ring finger- Associated (SRA) domain. The third family is made up of three related Zinc-finger proteins: Kaiso, ZBTB4, and ZBTB38.

The DNA methyltransferases DNMT1, DNMT3a, and DNMT3b are essential for mouse viability [4]. In contrast, the deletion of *Mbd1* [5], *Mbd2* [6], *Mbd4* [7], *Mecp2* [8], [9], or *Kaiso* [10], yields animals that are viable and fertile. The compound knockouts *Mbd2*/*Mecp2* and *Kaiso*/*Mbd2*/*Mecp2* have consequences similar to the single *Mecp2* knockout [11]. The only MBP that has been shown to be essential for development so far is UHRF1 [12].

There are at least three possible explanations for the lack of major phenotype seen upon deletion of MBD genes. First, it is possible that DNA methylation is essential, but that it does not act primarily by recruiting MBPs. It could instead serve mostly to inhibit the interaction of DNA-binding proteins with the genome [13], [14]. Second, it is possible that there is extensive redundancy between MBD proteins. Third, it is possible that other MBD proteins remain to be found. In support of this last possibility, a systematic search of the mammalian genome has uncovered 6 additional proteins with domains related to the MBD: BAZ2A (also called TIP5) and BAZ2B; the histone methyltransferases KMT1E (also called ESET or SETDB1) and KMT1F (also called CLLD8 or SETDB2); and two uncharacterized proteins, KIAA1461 and KIAA1887, that were renamed MBD5 and MBD6 [15], [16].

The mammalian genes MBD5 and MBD6 contain an intron within the MBD-coding region; this intron exists in the same location in the “canonical” MBDs (MBD1–4 and MeCP2), but is absent from BAZ2A, BAZ2B, SETDB1, and SETDB2 [15]. Therefore, from an evolutionary standpoint, MBD5 and MBD6 are more closely related to the well-characterized MBDs than BAZ2A, BAZ2B, SETDB1, and SETDB2. This is also supported by a phylogenetic analysis based on the amino-acid sequence of the MBD domain [15].

MBD5 is expressed in the human brain, and several lines of evidence link MBD5 mutations with mental disorders. First, a microdeletion of the *MBD5* gene has recently been shown to correlate with mental retardation in 8 human patients [17], [18], [19]. Additionally, 4 low-frequency missense variants in the coding sequence were found in one or more mentally retarded patients but not in healthy controls [18]. Finally, the *MBD5* gene is located on chromosome 2q23.1, a region in which aberrations are associated with epilepsy [20]. Mutations in *MECP2* cause Rett syndrome, a neurodevelopmental disorder [8], [9], and it is tempting to speculate, by analogy, that MBD5 is also a protein that binds methylated DNA and whose loss causes cerebral dysfunctions.

MBD6 is also expressed in the human brain, and it might be involved in neurodegenerative diseases for the following reasons. ATXN1 is an RNA-binding protein present in neuron nuclei; the expansion of its polyglutamine domain causes spinocerebellar ataxia type 1 (SCA1) [21]. ATXN1L is related to ATX1, with which it interacts, and it attenuates the neurotoxic effects of mutant ATXN1 [22]. It was found in a two-hybrid screen that ATXN1L interacts with MBD6 [23].

In this study, we have initiated the characterization of the human proteins MBD5 and MBD6. In particular, we have tested the hypothesis that they might bind methylated DNA. Our findings suggest that MBD5 and MBD6 associate with heterochromatin, and that their MBD is involved in this association, but that the proteins do not bind methylated DNA.

## Results

### Organization of the *MBD5* and *MBD6* genes and corresponding proteins

**Figure 1:**
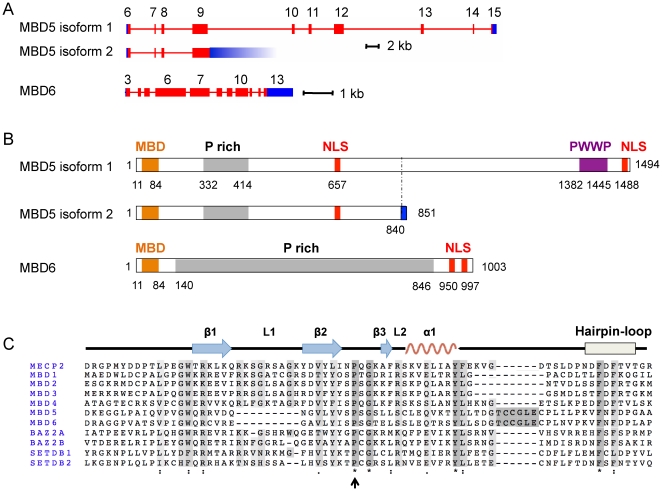
Organization of the human MBD5 and MBD6 genes and proteins. A- The coding exons of human *MBD5* (top) and *MBD6* (bottom). Translation of exons 6–15 of MBD5 yields protein isoform 1; translation of exons 6–9 with retention of the following intron yields protein isoform 2. B- Organization of the human MBD5 and MBD6 proteins. Amino-acid numbers are indicated. P rich: proline-rich segment; PWWP: PWWP domain; NLS: putative nuclear localization signal. The amino-acids that are unique to MBD5 isoform 2 are depicted in blue. C- Sequence comparison of all known human MBD domains. Periods, colons, and stars indicate increasing conservation of a given residue. The arrow shows the residue we mutated in MBD5 for further experiments. The secondary structure of MeCP2 is shown at the top. ß: Beta-sheet; α: alpha-helix; L: loop.

The human *MBD5* gene has 15 exons (Figure 1A). The Uniprot database describes two isoforms for MBD5 (Figure 1B): the longer variant, Isoform 1 (Q9P267-1), has 1448 amino-acids, and is encoded by exons 6 to 15; Isoform 2 (Q9P267-2) is encoded by exons 6 to 9, with the intron following exon 9 being retained. This results in a protein of 851 residues, with residues 1–841 shared with Isoform 1, and residues 842–851 unique to this isoform. We have been able to detect cDNAs corresponding to both isoforms, and we have observed in western blotting two MBD5 bands, with molecular weights consistent with the predicted isoforms (see following section). As for MBD6, the expression databases suggest that it is expressed mostly as one species, encoding a protein of 1003 amino-acids.

In each protein, the MBD is N-terminal (Figure 1B). Both MBD5 isoforms contain a stretch of 80 amino-acids that is Proline-rich (23 out of 80 residues are Proline). The C-terminus of Isoform 1 also contains a domain with a proline-tryptophan-tryptophan-proline (PWWP) central core. This domain is found (but not exclusively) in chromatin-associated proteins such as DNMT3A, DNMT3B, BRD1 and BRPF1. The central portion of MBD6, accounting for 70% of its total length, is Proline-rich (181 out of 706 residues are Proline). Finally, both MBD5 and MBD6 contain putative Nuclear Localization Signals (NLS) [24], and are therefore predicted to be nuclear proteins.

As pointed out in two earlier studies [15], [16], MBD5 and MBD6 present two major differences with other human MBDs: a deletion of 9 amino-acids in the first third of the MBD, and an insertion of 6 amino-acids in the last third (Figure 1C). The three-dimensional structure of the MBD from MBD1 [25] and MeCP2 [26] has been determined; 3 beta-sheets, an alpha-helix, and a hairpin-loop occur at identical positions in both proteins. The insertion and the deletion that occur in MBD5 and MBD6 are predicted not to disrupt any of these features, suggesting that their MBD may have an overall architecture similar to that of MBD1 and MeCP2.

### Detection of two MBD5 protein isoforms in cells

**Figure 2:**
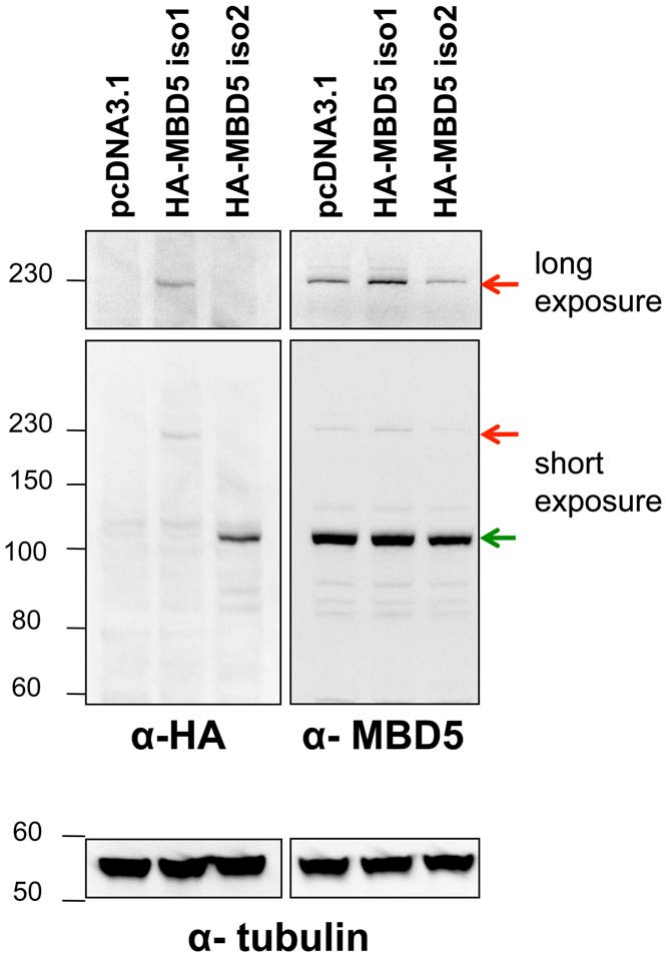
Detection of MBD isoforms 1 and 2 in cell extracts. Human MCF7 cells in culture were transfected with the indicated expression plasmids. Total cell extracts were probed, in parallel, with anti-HA antibodies to detect exogenous proteins, and with anti-MBD5 antibodies to detect both endogenous and exogenous proteins. Top: long exposure of the high-molecular weight region demonstrates the existence of Isoform 1. Middle: Isoform 2 is detected as a 110-kDa-migrating species. Bottom: detection of Tubulin proves equal loading between samples. The arrows indicate the two MBD5 isoforms; position of migration markers, with their indicative weight in kDa, is indicated.

We generated vectors for the expression of HA-tagged Isoform 1 or Isoform 2 of MBD5, which we transfected into human cells. Western blotting revealed that HA-Isoform 1 had an apparent molecular weight of 230 kDa, and HA-Isoform 2 an apparent molecular weight of 110 kDa (Figure 2). We raised rabbit polyclonal antibodies against MBD5, and used them to probe total extracts of human cultured cells by western blotting. We detected an intense band at 110kDa, which superimposes precisely with the HA-Isoform 2 band (the extra 10 amino-acids of the HA tag probably generate a molecular weight shift too small to be detected). There was also a less intense band at 230 kDa, superimposable with HA-Isoform 1. These results suggest that the two isoforms of MBD5 are indeed expressed in cultured cells.

### MBD5 and MBD6 are differentially expressed in mouse tissues; MBD5 Isoform 2 is highly expressed in oocytes

**Figure 3:**
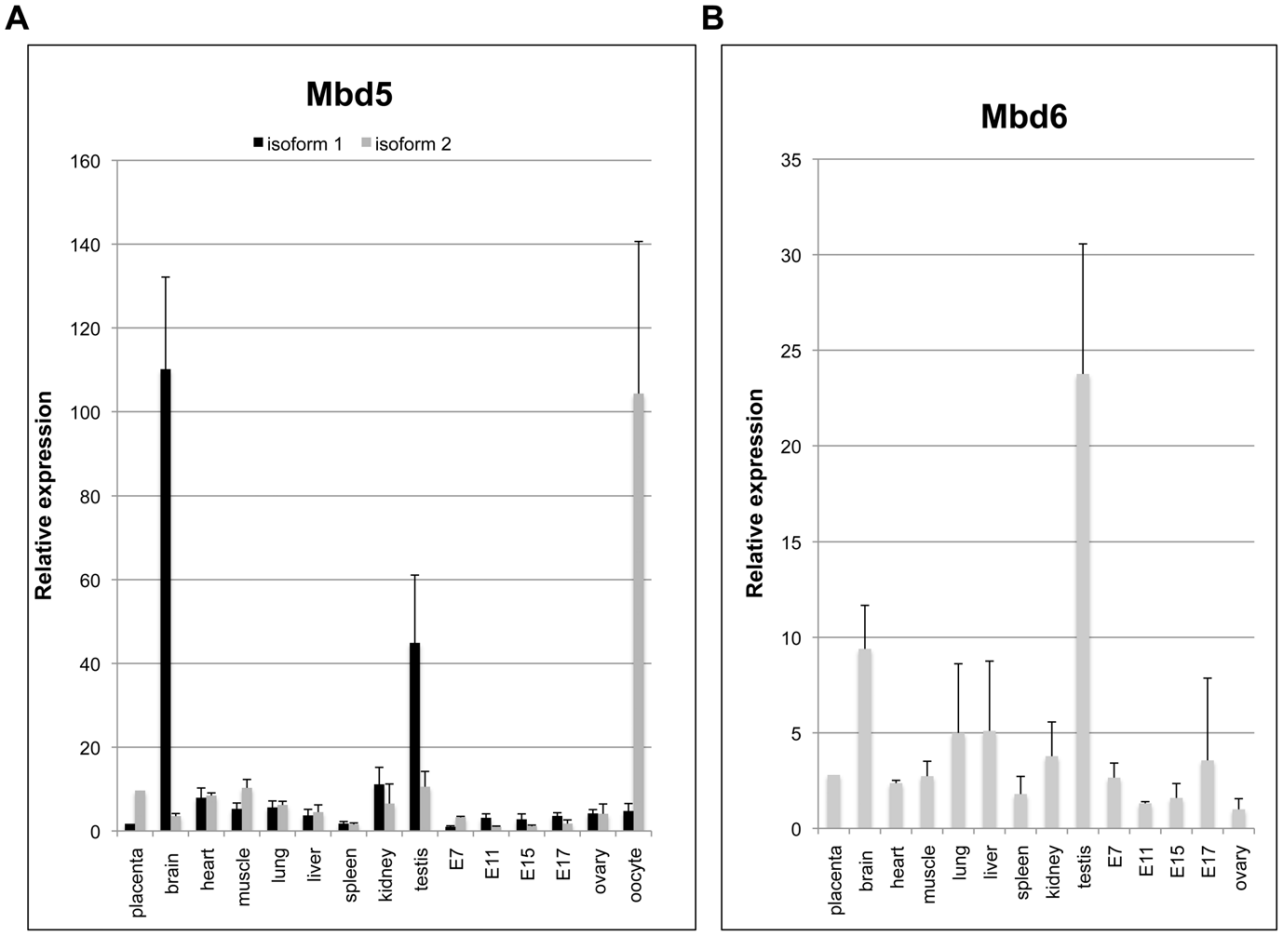
MBD5 and MBD6 are differentially expressed in mouse tissues. A-MBD5 Isoform 1 is expressed at highest levels in the brain, whereas isoform 2 is most expressed in oocytes. B- MBD6 is expressed at highest levels in the testis. Expression levels were measured by quantitative RT-PCR, with normalization to the RPS16 gene.

We then sought to identify the expression pattern of MBD5 and MBD6. For this, we quantified their expression in a variety of mouse tissues by quantitative RT-PCR (Figure 3). Two pairs of primers were used for MBD5: one spans the exon 14-exon 15 junction and is specific for Isoform 1. The other spans exon 9 and the following intron; it detects Isoform 2 specifically (See Table 1 for primer sequences). We found that Isoform 1 was expressed in all tissues, but with a wide range of levels: the lowest levels were seen in E7 embryos (Embryos at day 7 of development), and the highest levels in the brain (110-fold higher than E7 embryo) and testis (45-fold higher than E7 embryo). Isoform 2 is conspicuously different: its level is relatively homogeneous in the tissues we tested, but it is very high in oocytes (100-fold higher than in E11 embryos, the sample with lowest expression). These observations agree with previous reports of MBD5 expression in the brain, as well as with data present in public databases (BioGPS, http://biogps.gnf.org/; MBD5 isoform 1: probe 1456423_at; MBD5 isoform 2: probe gnf1m21841_at; MBD6: probe gnf1h08707_at).

**Table 1:** qRT-PCR primers used in this study.

Target	Forward sequence	Reverse sequence
Mbd5 Isoform 1	GAGGCCATGAGCGAACTG	TCTTCCTCCTCTTGGGTTTG
Mbd5 isoform 2	ACGTCCTCCACTCCAGTGAT	TTCACAATGGGGAAAGGAAC
Mbd6	CCCGGGGATAGTCAGAAAGT	AGCTGCTCGCGTTGTAGG
RPS16	AGGAGCGATTTGCTGGTGTGG	GCTACCAGGGCCTTTGAGATG

We also screened for MBD6 expression. The cDNA was detected in all tissues, with a range of expression more narrow than for MBD5: there was a 24-fold difference between the highest-expressing tissue (testis), and the lowest-expressing tissue (ovary).

We note that MBD5 and MBD6 are highly expressed in organs where epigenetic reprogramming occurs: in the testis and in oocytes.

### MBD5 and MBD6 can localize to methylated loci; this requires the MBD

**Figure 4:**
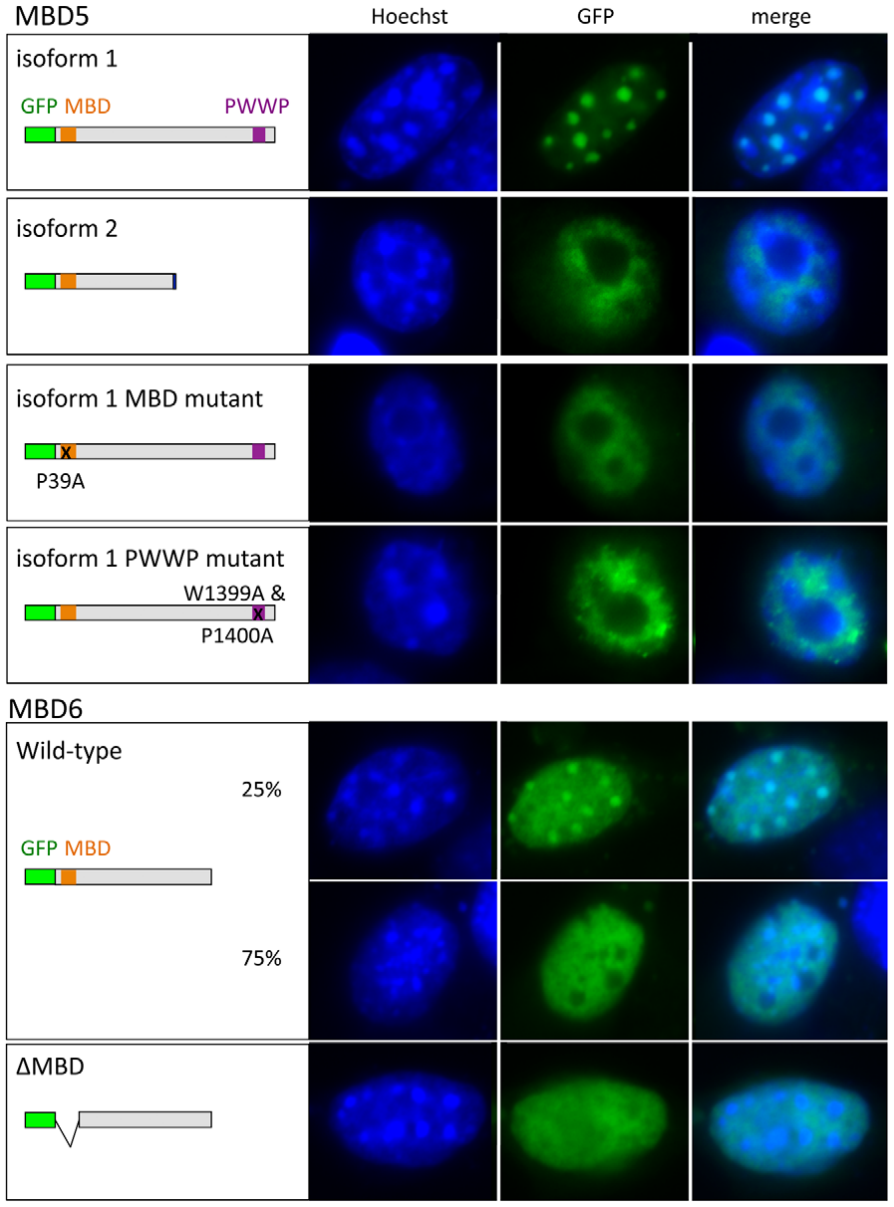
MBD5 and MBD6 can colocalize with methylated regions in mouse nuclei. The indicated proteins were transfected into mouse 3T3 cells. The distribution of the various proteins in fixed cells was recorded, and representative images are provided. When different types of localization occurred, their approximate proportion is indicated. A-MBD5 isoform 1 always colocalizes with the chromocenters, DAPI-dense regions that harbor hypermethylated heterochromatin; mutating either MBD or PWWP domain changes this distribution to a diffuse nuclear pattern. Isoform 2 is also incapable of chromocenter localization. B- MBD6 colocalizes with chromocenters in 25% of the observed cells. Upon deletion of the MBD, the pattern becomes diffuse in all cells.

When expressed in mouse cells, most methyl-binding proteins are found in the pericentric heterochromatin, a compartment made up of heavily methylated repeats [27], [28], [29]. Cytologically, this compartment is easily recognizable: it stains brightly with DAPI or Hoechst-33342 because of its AT-rich base composition. For this reason, we sought to determine the intracellular localization of the two MBD5 isoforms, and of MBD6, upon transfection in mouse cells. The proteins were tagged with enhanced Green Fluorescent Protein (GFP) at their N-terminus, and expressed in NIH-3T3 cells (Figure 4).

We observed that MBD5 (both isoforms) and MBD6 are nuclear proteins. Isoform 1 of MBD5 was always found at the chromocenters, whereas isoform 2 never was (Figure 4A). To identify the determinants necessary for chromocentric localization, we introduced inactivating point mutations in the MBD or the PWWP domains of MBD5 Isoform 1. Mutating either domain resulted in a complete loss of chromocentric colocalization: the mutant proteins showed a diffuse nuclear staining. Therefore both the MBD and PWWP domains are necessary, but neither are sufficient, for recruitment of MBD5 Isoform 1 to the methylated pericentric heterochromatin. MBD6 displayed a heterogeneous subnuclear localization in the cell population: in a quarter of the cells the protein overlapped with the chromocenters; in the remaining cells the protein diffused homogeneously within the nucleus (Figure 4B). We sought to introduce a point mutation in the MBD of MBD6, but our multiple attempts at inverse PCR were unsuccessful, probably as the result of the unusual sequence characteristics of the MBD6 cDNA. We succeeded, however, in deleting this domain. The resulting truncated protein was still nuclear, but was never enriched at chromocenters, indicating that the MBD is necessary for recruitment to the pericentric heterochromatin.

These results show that MBD5 and MBD6 can be recruited to the highly methylated pericentric heterochromatin of mouse cells, and that this function requires their respective MBDs. This finding is compatible with the possibility that these domains bind methylated DNA.

### MBD5 and MBD6 can localize at the chromocenters independently of Dnmt1

We then sought to find out whether the localization of MBD5 and MBD6 at chromocenters required their containing methylated DNA. Mouse fibroblasts lacking DNMT1 have been established in a p53-null background [30]; their chromocenters are undermethylated to varying levels in the cell population and, in many cells, the hypomethylation is sufficient to prevent recruitment of methyl-binding proteins.

**Figure 5:**
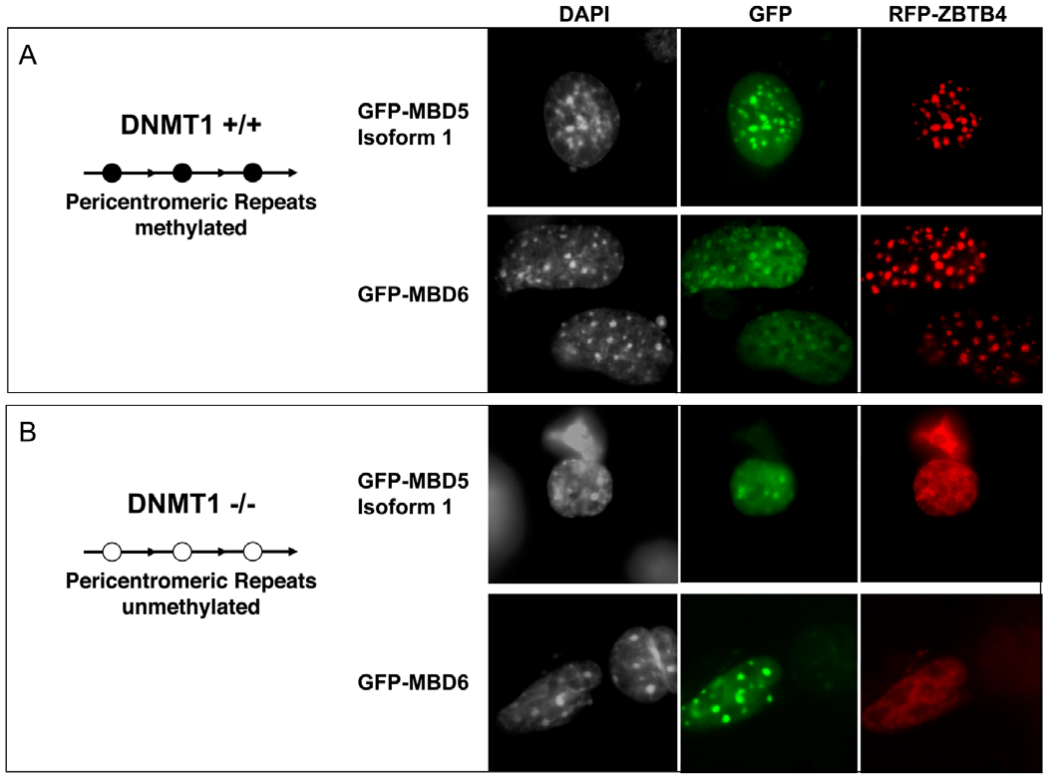
MBD5 and MBD6 can be recruited to chromocenters in *Dnmt1*−/− cells. The various GFP fusions were transfected into mouse fibroblasts of the indicated genotype. A -ZBTB4, MBD5, and MBD6, are recruited to the chromocenters of *Dnmt1*+/+ cells. B- MBD5 and MBD6 can be recruited to the demethylated chromocenters of *Dnmt1*−/− cells; ZBTB4 does not overlap with the chromocenters in the same cells.

We transfected GFP fusions of MBD5 and MBD6 into *Dnmt1*−/− cells and matching control *Dnmt1*+/+ cells (Figure 5). We also included in the transfection an RFP-ZBTB4 expression construct: as we have previously reported, this protein does not associate with chromocenters in cells that have lost methylation [29].

In the *Dnmt1*+/+ cells, we observed a situation identical to that reported above for 3T3 cells: MBD5 was always associated with chromocenters, whereas MBD6 was only associated with the chromocenters in about 25% of transfected cells. ZBTB4 was always associated with chromocenters (Figure 5A).

We then examined *Dnmt1*−/− cells. The diffuse nuclear localization of RFP-ZBTB4 (as opposed to chromocenter association) was used to ensure that the transfected cells indeed had a low level of DNA methylation. In the cells with diffuse ZBTB4, we observed that MBD5 was still associated with chromocenters. Similarly, MBD6 sometimes colocalized to chromocenters, even in cells where ZBTB4 was delocalized.

These results indicate that MBD5 and MBD6 can associate with chromocenters even in cells where the DNA is demethylated enough to prohibit recruitment of ZBTB4. This could mean that MBD5 and MBD6 bind methylated DNA *in vivo* with an affinity greater than that of ZBTB4. Alternatively, it could mean that MBD5 and MBD6 are attracted to pericentric heterochromatin by a determinant that does not depend on DNA methylation.

### The MBD domains of MBD5 and MBD6 do not bind methylated DNA *in vitro*

**Figure 6:**
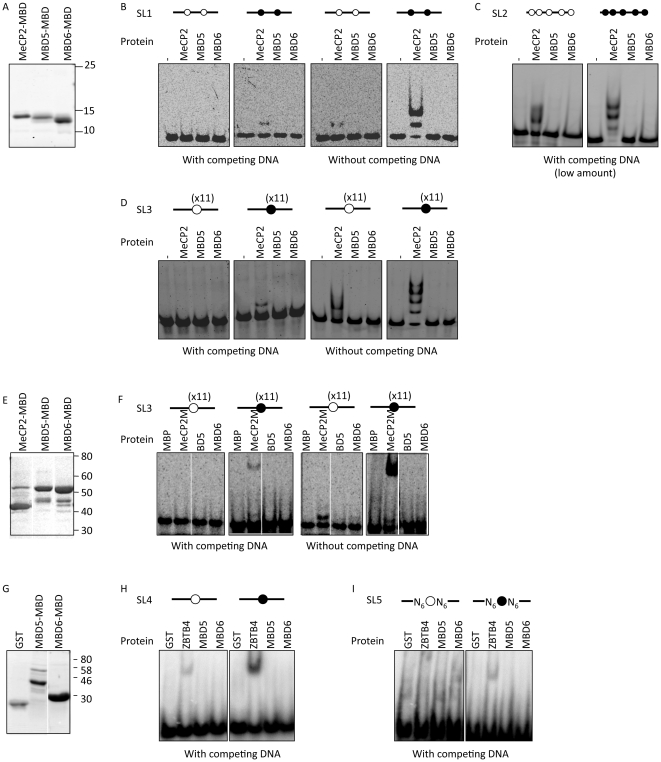
The MBD domain of MBD5 and MBD6 does not bind methylated DNA *in vitro*. A- The MeCP2, MBD5 and MBD6 MBDs, tagged with 6×His, were expressed in bacteria, purified, and examined on a Coomassie-stained SDS-PAGE gel. Apparent weights of migration markers, in kDa, are indicated. B-Gel retardation assay with probe SL1 and the proteins of panel A. In this panel and the following, the probe is depicted as a line; open circles represent an unmethylated CpG, and filled circles a methylated CpG. In panels 2B–2D, the dash indicates the probe-only lane (no protein added). “MeCP2” : MBD domain of MeCP2, tagged with 6×His; same abbreviation for MBD5 and MBD6. C-Gel retardation assay with probe SL2 and the proteins of panel A. D-Gel retardation assay with probe SL3 and the proteins of panel A. E- The MeCP2, MBD5 and MBD6 MBDs, tagged with Maltose-Binding Protein (MBP), were expressed in bacteria, purified, and examined on a Coomassie-stained SDS-PAGE gel. F-Gel retardation assay with probe SL3, and the proteins of panel E. “MeCP2” : MBD domain of MeCP2 tagged with MBP; same abbreviation for MBD5 and MBD6. “MBP” : Maltose-Binding Protein only lane. G- The MBDs of MBD5 and MBD6, tagged with GST, were expressed in bacteria, purified, and examined on a Coomassie-stained SDS-PAGE gel. A region of MBD5 larger than just the MBD was included in this experiment, explaining the higher molecular weight. H-Gel retardation assay with probe SL4. “ZBTB4”: Zinc fingers of ZBTB4 fused to GST; “MBD5” : MBD domain of MBD5 fused to GST, same abbreviation for MBD6. “GST”: GST-only lane. I-Gel retardation assay with probe SL5 and the proteins used in panel H. Probe SL5 contains 6 randomized positions (“N”), on either side of the CpG. Legend as in panel H.

To assess the DNA binding properties of MBD5 and MBD6 *in vitro* we performed Electrophoretic Mobility Shift Assay (EMSA) experiments with oligonucleotides containing cytosines that were unmethylated or methylated. We carried out 7 different experiments, using various combinations of recombinant proteins and DNA probes. The MBD domain of human MeCP2 (AA 77–164) was used as the positive control in most experiments (the exception, presented in figure 6H and 6I, is explained below), and we investigated the homologous regions of human MBD5 and MBD6 (AA 1–93 of each protein).

The first 3 experiments used proteins tagged with 6 Histidines at the N-terminus (6×His tag). All proteins were equally pure and soluble, and they were used at equal molar amounts (Figure 6A). Experiment 1 employed probe SL1, an artificial sequence that contains 2 CpGs (Probe sequences are given in Table 2). We observed, as expected, that MeCP2 bound the methylated probe, but not the unmethylated version of the same probe (Figure 6B). Under the same conditions, neither MBD5 nor MBD6 bound probe SL1. This experiment was carried out under standard EMSA conditions: it included non-specific competitor DNA (poly dA–dC). We hypothesized that these conditions might mask a positive result if MBD5 and MBD6 bind DNA non-specifically. To test this possibility, we repeated the binding experiments in the absence of competitor. Under these conditions, as expected, MeCP2-MBD interacted non-specifically with DNA: it shifted both methylated and unmethylated probes. In contrast, both MBD5 and MBD6 failed to shift the probes (Figure 6B).

**Table 2:** Oligonucleotides used for EMSA analyses. The reverse strand is not shown; at the underlined positions either cytosine (for unmethylated probes), or 5-methyl-cytosine (for methylated probes), was incorporated during synthesis of the oligonucleotides. The only exception is probe SL5, which was methylated *in vitro*, using SssI.

SL1	5′-TAGTGCCCTCCGCCATGTACCATGGATCCGATGTACAT-3′
SL2	5′-TAGACATTGCCCTCGAGGTACCATGGATCCGATGTCGACCTCAAA CCGAGACGAATTCCG-3′
SL3	5′-CGCGACGACGCACCACGACGCACGACGCGAACGCGCCAA-3′
SL4	5′-GTAGAAGTTCCCGCCATCACTATTG-3′
SL5	5′- GTTTTCCCAGTCACTAC(N_6_)CG(N_6_)GTCATAGCTGTTTCCTG -3′

We then used the same proteins to carry out an EMSA experiment in the presence of a limited amount of competitor DNA, and with an unrelated probe, SL2, that contains 5 CpGs (Figure 6C). Identical results were obtained: MeCP2 bound the methylated probe, and, with less efficiency, the unmethylated probe. MBD5 and MBD6 failed to bind either probe.

To increase the probability of detecting an interaction between MBD5, MBD6, and DNA, we then moved on to a probe with a very high CG proportion, SL3: it contains 11 CpG dinucleotides (Figure 6D). MeCP2 bound the methylated probe, but not the unmethylated probe. MBD5 and MBD6 did not bind the probe in either condition. And, again, the removal of competitor DNA failed to uncover an interaction between MBD5 or MBD6 and DNA.

We considered the possibility that the 6×His tag interfered with the function of MBD5 and MBD6. To investigate this, we changed to a different tag, Maltose-Binding Protein (MBP). MBP-MBD5, and MBP-MBD6 were highly expressed and soluble; MBP-MeCP2 was expressed less efficiently (Figure 6E); the three proteins were used at the same concentration to test interaction with the CpG-rich probe SL3. MBP-MeCP2 displayed methylation-dependent interaction with the probe; MBP-MBD5 and MBP-MBD6 did not interact with either methylated or unmethylated probe, and also failed to significantly bind the probe in the absence of competitor DNA (Figure 6F).

Our third tagging approach was to fuse the MBD domains to GST (Figure 6G). Under these conditions we first tested probe SL4. It contains a single methylated CpG, that can be recognized by Kaiso, ZBTB4, and ZBTB38 [31]. GST-ZBTB4 was the positive control in this experiment, and it bound methylated SL4. GST-MBD5 and GST-MBD6 were inactive (Figure 6H).

It is possible that MBD5 and MBD6 have specific sequence requirements for binding, and that these requirements are unfulfilled in any of the probes investigated so far. Therefore we turned to probe SL5: it is a mixture of oligonucleotides that all contain a fixed central CpG flanked by six randomized positions on either side. ZBTB4, which binds methylated DNA in a sequence-specific fashion, interacted with some of the labeled oligonucleotides. In contrast, GST-MBD5 and GST-MBD6 did not show any detectable interaction with probe SL5 (Figure 6I).

We conclude that, *in vitro*, MBD5 and MBD6 do not bind the different methylated sequences that were tested.

## Discussion

### MBD5 and MBD6 do not bind methylated DNA *in vitro*

We find that MBD5 and MBD6 do not bind methylated DNA *in vitro*. As with any negative conclusion, this has to be carefully qualified.

First, it is possible that the proteins expressed in bacteria are not correctly folded or lack a critical post-translational modification. One argument against this possibility is that the MBD of MeCP2, expressed in parallel using the same systems, was functional in our assays. In addition, a large number of published data show that bacterially expressed MBD1, MBD2, MBD4, and MeCP2 are active for binding methylated DNA [25], [28]; this establishes that their MBD need not be post-transcriptionally modified to be active, and argues that the same is likely to hold for MBD5 and MBD6. Nevertheless, the identification of a ligand that binds MBD5 or MBD6, and that could be used as a positive control for their activity *in vitro*, will be necessary to formally rule out the possibility that the bacterially produced proteins are inactive.

A second theoretical possibility is that MBD5 and MBD6 bind methylated cytosine in a very particular sequence context, which was not present in the probes we used. Again, this seems unlikely for the two following reasons. First, MeCP2 favors certain binding sites *in vitro* [32], but changing the ratio of protein, probe, and non-specific competitor can easily reveal binding to suboptimal sites; placing multiple CpGs on a probe also easily overrides the sequence specificity. An illustration of this is provided by the fact that none of the probes used here contain optimal MeCP2 binding sites, yet they were clearly bound *in vitro*. By analogy, if MBD5 and MBD6 were methyl-binding proteins with specific sites, it seems likely that we should have detected some binding under the rather relaxed conditions that were used. Second, we have included in our tests a randomized probe, which is sufficiently complex to permit binding of ZBTB4, a methyl-binding protein that requires a defined consensus around the methylated CpG [31].

The simplest explanation for our results is that MBD5 and MBD6 do not bind methylated DNA. This possibility had been predicted by Hendrich and Tweedie based upon sequence examination [15]. We concur with their idea that this behavior results, at least in part, from the 9 amino-acid deletion that removes a region homologous to loop L1 of MBD1 and MeCP2. In these two proteins, loop L1 enters the major groove of DNA and interacts with the DNA backbone; it is critical for recognition of methylated DNA [25], [26].

At least one other MBD protein does not bind methylated DNA: MBD3 [27]. Interestingly, this loss of function is rather recent in the course of evolution, as Xenopus MBD3 does bind methylated DNA, whereas mammalian MBD3 does not [33]. Is it possible that MBD5 and MBD6 also recently lost their methyl-binding activity? Database searches readily reveal proteins containing MBDs related to that of MBD5 and/or MBD6 in mammals, non-mammalian vertebrates (including Xenopus and Zebrafish), as well as invertebrate animals (including Amphioxus and insects). In all of these cases, the MBDs have the same insertion and deletion as human MBD5 and MBD6. Our prediction, therefore, is that MBD5 and MBD6 are not methyl-binding proteins in other species either.

### How are MBD5 and MBD6 recruited to pericentric heterochromatin

We report that MBD5 and MBD6 can be recruited to pericentric heterochromatin even in dnmt1−/− mutant cells. It could be argued that the residual DNA methylation existing in the chromocenters of dnmt1−/− cells is sufficient to attract MBD5 and MBD6, however this would imply that both proteins have a higher affinity for methylated DNA than ZBTB4 and several other previously known MBPs — a possibility difficult to reconcile with our *in vitro* results. A simpler explanation would be that MBD5 and MBD6 are recruited to chromocenters by a component of heterochromatin other than methylated DNA. Three hypotheses are attractive. First: an interaction with RNA. The MBD-containing protein BAZ2A/TIP5 is recruited to methylated rDNA repeats by an RNA [34]. The chromocenters produce non-coding RNA [35], [36], so it is possible that a similar mechanism is at work here. Another possibility would be the interaction with modified histones: H3K27me1, H3K9me3, and H4K20me3 are enriched in chromocenters [37], and the PWWP domain has been shown in certain proteins to recognize modified histones [38], [39].

### MBD5 and MBD6: potential roles in epigenetic reprogramming

MBD5 and MBD6 associate with heterochromatin, and it is tempting to speculate that they play a role in the epigenetic regulation of cellular identity. We have found that MBD5 and MBD6 are expressed at high levels in a few tissues, including brain and testis. It might be that MBD5 and MBD6 contribute to the unique epigenetic machinery of neurons or to the global reorganization of chromatin during spermatogenesis [40], [41].

Along similar lines, we report that Isoform 2 of MBD5 is expressed at very high levels in mouse oocytes. This expression pattern is reminiscent of other proteins, including Stella, that play important roles in the epigenetic remodeling that occurs after fertilization [42], [43]. If this specific isoform of MBD5 is also involved in this process, female *Mbd5* mutants might be sterile. This prediction will have to be tested by experimental work, including the examination of *Mbd5−/−* mouse, which are currently being generated.

## Materials and Methods

### Plasmids

A cDNA clone containing the Isoform 1 of human MBD5 was obtained from Origene (Clone reference: SC113547). To obtain isoform 2, we PCR-amplified this clone with two nested primers adding the 11 amino-acids that are specific to isoform 2. A cDNA clone for human MBD6 was obtained from ATCC (Clone reference: 10437624). Site-directed mutagenesis was performed by inverse PCR. For imaging, MBD5 (both isoforms), MBD6, and their mutant derivatives were cloned into peGFP-C2 (Clontech). The mutants were sequenced, and we verified by western blotting that the proteins expressed had the expected size. HA-tagged versions for expression in mammalian cells were constructed by nested PCR and cloned into pcDNA 3.1 (Invitrogen).

For bacterial protein expression, clones encoding the MBD of MBD5 and MBD6 were generated by assembly of oligonucleotides with optimized codons for expression in *E. coli*; they were cloned into pET21a (Novagen), and bear an N-terminal 6×His tag. We additionally cloned the MBD of MBD6 and MBD6 into an MBP fusion plasmid (pMALp2X, New England Biolabs) and a GST fusion fusion plasmid (pGEX-5X-1, GE Heathcare). The clone encoding the MBP fusion of MBD of MeCP2 and His-tagged MBD domain of MeCP2 were a kind gift of Drs. Priscilla Too and Shuang-Yong Xu, respectively (New England Biolabs).

New England Biolabs supplied all the enzymes and their buffers, protein and DNA markers, plasmids and competent cells. All PCR used Phusion Hot start (Finnzyme, Finland). Plasmids were purified with Qiaprep® spin columns (Qiagen, USA), and PCR products with the Wizard®SV Gel and PCR Clean-Up System (Promega, USA). All plasmid names and origins are given in Table 3.

**Table 3:** Plasmids used in this study.

Name	Description	Source
pET21a	Vector for bacterial expression of proteins	Novagen
pEGFP-C2	Mammalian expression vector for EGFP	Clontech
pCDNA3.1	Mammalian expression vector	Invitrogen
PAD665	ZBTB4 cloned into pmRFP-C2	Filion 2006
PAD1358	Human MBD5 cDNA (clone SC113547)	Origene
PAD1359	Human MBD6 cDNA (clone 10437624)	ATCC
PAD1360	MBD5 Isoform 1 cloned into pEGFP-C2	This study
PAD1361	MBD5 isoform 2 cloned into pEGFP-C2	This study
PAD1362	MBD5 Isoform 1 with MBD mutation cloned into pEGFP-C2	This study
PAD1363	MBD5 Isoform 1 with PWWP mutation cloned into pEGFP-C2	This study
PAD1364	HA-tagged MBD5 Isoform 1 cloned into pCDNA3.1	This study
PAD1365	HA-tagged MBD5 isoform 2 cloned into pCDNA3.1	This study
PAD1366	MBD6 cloned into pEGFP-C2	This study
PAD1367	MBD6 with deletion of the MBD cloned into pEGFP-C2	This study
PAD1368	Bacterial expression vector for 6×His-tagged MBD of MBD5	This study
PAD1369	Bacterial expression vector for 6×His-tagged MBD of MBD6	This study
PAD1370	Bacterial expression vector for Maltose-Binding-Protein-tagged MBD of MBD5	This study
PAD1371	Bacterial expression vector for Maltose-Binding-Protein-tagged MBD of MBD6	This study
PAD1372	Bacterial expression vector for GST-tagged MBD of MBD5	This study
PAD1373	Bacterial expression vector for GST-tagged MBD of MBD6	This study

### Reverse transcription and quantitative PCR

Work with mice and the corresponding protocols have received the agreement # 5314 from Ministère de l'Enseignement Supérieur et de la Recherche (Paris, France). We extracted RNAs from mouse placenta and ovaries using Trizol (Invitrogen), and performed reverse-transcription with Superscript III (Invitrogen) and oligo-dT. Oocyte cDNAs were prepared as described previously [44]. We verified that our cDNA preparations were not contaminated by genomic DNA by performing qPCR in the absence of reverse transcription. cDNAs isolated from the other mouse tissues were purchased from Clontech, and were tested for contamination by the manufacturer. qRT-PCR primer sequences are in Table 1. We verified that they gave linear amplifications, and we measured values only within the validated range. In each sample we measured the abundance of the housekeeping gene RPS16, and normalized the data using the 2̂-(ΔΔCt) method [45]. The tissue with lowest expression of MBD5 or MBD6 was arbitrarily set to a value of 1, to permit easier comparisons. The error bars in figure 3 represent the standard deviation between three technical replicates in one representative experiment. All experiments were carried out at least three times. Placental cDNA was included in all the experiments as an internal control.

### Antibodies and western-blotting

The MBD5 rabbit polyclonal antibody was raised against several peptides of the human protein. For western-blotting, the purified antibody was used at a dilution of 1∶1000 overnight at 4°C in PBST-5% milk, followed by standard washing and revelation steps.

### Transfection and microscopy

NIH-3T3, *p53*−/−, and *p53*−/−;*Dnmt1*−/− MEFs were grown on coverslips in 24-well plates with DMEM/10% FBS, and were transfected using Lipofectamine 2000 (Invitrogen). To observe GFP, the cells were collected twenty-four hours after transfection, rinsed with PBS, fixed with 2% paraformaldehyde for 10 minutes, permeabilized with 0.5% Triton X-100 for 4 minutes, then DNA was stained with Hoechst 33342 or DAPI.

### Protein expression and purification

T7 Express fresh transformants of *E. coli* were grown on agar plates overnight and used to inoculate a 10 mL preculture, which was then used to start a full-size culture (generally 1 liter). The culture was incubated at 37°C until the Optical Density at 600 nm reached 0.6, then expression was induced with 0.5 mM IPTG at 16°C overnight. After this the cells were pelleted and resuspended in lysis buffer (20 mM Tris-HCl pH 8, 500 mM NaCl, 5% glycerol, 10 mM Imidazole, 0.1% Triton X-100, 10 mM β-mercaptoethanol and 1 mM PMSF). The cells were lysed by sonication, the lysate clarified, and the supernatant applied to Ni-NTA beads (Qiagen) preequilibrated with 5 bed volumes of Histag binding buffer (20 mM Tris-HCl pH 8, 500 mM NaCl, 5% glycerol, 10 mM Imidazole). After washing, proteins were eluted using 2 bed volumes of the Histag elution buffer (20 mM Tris-HCl pH 8, 500 mM NaCl, 5% glycerol, 200 mM Imidazole, 10 mM DTT). Proteins were concentrated and buffer exchanged using a Millipore 3,000 MWCO spin column into the heparin column binding buffer (20 mM Tris-HCl pH 8, 0.1 mM EDTA, 5% glycerol, 1 mM DTT). Heparin purifications were performed using Fast Performance Liquid Chromatography (FPLC) system and prepacked HiTrap™ Heparin resin (Amersham, UK). While the MeCP2-MBD protein eluted around 500 mM NaCl, the MBD of both MBD5 and MBD6 was collected in the flowthrough. After purification, proteins were stored at −20°C in a 50% glycerol storage buffer. To prepare GST and MBP proteins, we used similar steps. The proteins were purified in one step using GST and MBP resin respectively.

### Gel retardation assay

The sequences of the oligonucleotides used are given in Table 2. Probes SL1, SL2, and SL3, were labeled at the synthesis step with the fluorescent marker FAM. Probes SL4 and SL5 were radiolabeled.

In a typical reaction, 1 nM of probe was incubated for 20 min at 25°C with 1 µM of protein in the following binding buffer: 50 mM TrisHCl pH 8, 1 mM EDTA, 1 mM DTT, 1 mM PMSF, 5% glycerol. For EMSA in panel 6B, C, D and F, non-specific competitor DNA was poly(dAdC) (6B: 200 ng, 6C: 500 ng, 6D, 6F: 1 ug). In panel 6H–6I, we used 1 ug of poly(dIdC). Protein-DNA complexes were analyzed on 6% polyacrylamide TBE gels run in 0.5 TBE at 4°C for 1 hour at 100 V. Images were acquired with a Typhoon imager (for fluorescent probes), or a Phosphor-Imager (for radioactive probes).
